# Alterations in the immune system persist after one year of convalescence in severe COVID-19 patients

**DOI:** 10.3389/fimmu.2023.1127352

**Published:** 2023-02-13

**Authors:** Judith Abarca-Zabalía, Adela González-Jiménez, Myriam Calle-Rubio, Andrea R. López-Pastor, Tomás Fariña, Carlos Ramos-Acosta, Eduardo Anguita, Elena Urcelay, Laura Espino-Paisán

**Affiliations:** ^1^ Laboratorio de Investigación en Genética de Enfermedades Complejas, Instituto de Investigación Sanitaria del Hospital Clínico San Carlos (IdISSC), Madrid, Spain; ^2^ Department of Pneumology, Hospital Clínico San Carlos, Instituto de Investigación Sanitaria del Hospital Clínico San Carlos (IdISSC), Madrid, Spain; ^3^ Intensive Care Unit, Hospital Clínico San Carlos, Madrid, Spain; ^4^ Department of Medicine, Universidad Complutense de Madrid (UCM), Madrid, Spain; ^5^ Hematology Department, Instituto de Medicina de Laboratorio, Instituto de Investigación Sanitaria San Carlos (IdISSC), Hospital Clínico San Carlos, Madrid, Spain

**Keywords:** COVID-19, SARS-CoV-2, NK cells, immune system, activation, convalescence

## Abstract

**Introduction:**

Severe COVID-19 originates a myriad of alterations in the immune system during active disease, especially in the T and NK cell compartments, but several studies in the last year have unveiled some alterations that persist in convalescence. Although most of the studies follow the participants for a short recovery time, studies following patients up to three or six months still find alterations. We aimed at evaluating changes in the NK, T and B cell compartments after severe COVID-19 in participants with a median recovery time of eleven months.

**Methods:**

Eighteen convalescent of severe COVID-19 (CSC), 14 convalescent of mild COVID-19 (CMC) and nine controls were recruited. NKG2A, NKG2C, NKG2D and the activating receptor NKp44 were evaluated in NK^bright^, NK^dim^ and NKT subpopulations. In addition, CD3 and CD19 were measured and a basic biochemistry with IL-6 levels was obtained.

**Results:**

CSC participants showed lower NK^bright^/NK^dim^ ratio, higher NKp44 expression in NK^bright^ subpopulations, higher levels of serum IL-6, lower levels of NKG2A^+^ T lymphocytes and a trend to a lower expression of CD19 in B lymphocytes compared to controls. CMC participants showed no significant alterations in the immune system compared to controls.

**Conclusions:**

These results are concordant with previous studies, which find alterations in CSC weeks or months after resolution of the symptoms, and point to the possibility of these alterations lasting one year or more after COVID-19 resolution.

## 1 Introduction

In 2020, the outbreak of the SARS-CoV-2 pandemics posed a challenge to the scientific community. This novel virus can affect the human host in a variety of ways from the most frequent asymptomatic or mild cases with common cold or influenza-like symptoms, to severe acute distress, complicated bilateral pneumonia or a cytokine storm that can lead to death. Immunological alterations during active Coronavirus disease-19 (COVID-19) have been consistently reported; specifically lower cell counts of T and NK lymphocytes and higher levels of inflammatory markers such as IL-6 (1–3). NK cells captured a special interest due to their role in viral-infection control. A seminal study in mice unveiled the potential of NK cells to interact with CD4^+^T cells and modulate their response against a viral infection (4), while another study reported a similar role related to CD8^+^ T cells (5). Therefore, these cellular populations have a dual role as regulators of the immune response as well as promoters of inflammation, depending on the perceived stimuli.

Studies focused on NK cells in active COVID-19 found higher numbers of NKG2C^+^ cells in the severe forms of the disease (3). These cells, termed adaptive NK cells or memory NK cells, proliferate in response to cytomegalovirus and other viral infections (6, 7). In severe active COVID-19, NK cells expressed higher levels of NKp44 after activation with IL-2 (8, 9). NKp44 is one of the Natural Cytotoxicity Receptors (NCRs), a group integrated also by NKp30 and NKp46. While NKp30 and NKp46 are constitutively expressed in resting NK cells, NKp44 expression occurs only after NK cell activation (10). Imbalanced NK cell subpopulations were detected in subjects undergoing SARS-CoV-2 infection, with the NCAM1^+^CD160^+^ subpopulations being more prevalent and correlated with disease severity (11). Imbalances in NKG2A and NKG2D receptors, cell markers classically expressed by NK cells, have been also related to COVID-19 severity (3, 12).

During 2021, some studies evaluated the recovery of the immune system in the COVID-19 convalescent phase. The first studies defined convalescence as a very short time, between weeks and a month after hospital discharge (13, 14), but recently interesting results considered longer periods (15–19). In Chinese and German populations, alterations in the invariant NKT (iNKT) compartment were found up to two months after COVID-19 resolution, with increased apoptosis and lower numbers of iNKT cells (16). Patients followed up to six months after COVID-19 infection evidenced alterations in the T and B cell compartments. Some of these alterations resolved during the six months of convalescence, but some others, such as a higher cytotoxic profile in CD8^+^T cells, persisted after that time (19). Lower expression of the CD19 receptor and other changes in the B cell compartment were observed up to three months after hospital discharge (15). Interestingly, other authors did not find significant sequelae six months after resolution of the disease in 13 recovered mild-COVID patients and only one severe patient (17). This absence of alterations, when other studies have reported modified immune populations in convalescent patients of severe COVID-19, could suggest that people who experienced mild disease suffer reduced perturbations in the immune system and recover faster to a previous healthy state. Therefore, differences in the recovery of mild and severe COVID-19 patients seemed worth exploring. We aimed to characterize some of the alterations that persist in the NK cell compartment in subjects recovered from both severe and mild COVID-19. To this purpose, we selected NK cell markers that singled-out populations reportedly distorted during active disease.

## 2 Methods

### 2.1 Patients

Eighteen convalescent of severe COVID-19 (CSC) subjects were recruited months after resolution of the symptoms and were matched by age and sex with 14 individuals that had experienced mild COVID-19 (CMC). Known comorbidities reported to increase the risk of severe COVID-19, such as hypertension, cardiovascular disease, hypothyroidism or high body mass index precluded recruitment. CSC patients underwent intensive care (ICU) with mechanical respiratory support during their hospital stay, and did not have persistent symptoms of the disease or a diagnosis of long-COVID-19 at the moment of sample collection. Participants in this group, with a mean age at sample collection of 54 ± 8.84 years, were recruited on average between 10 and 12 months after ICU admission (13 of the 18 patients), with recovery time spanning from four and five months for two participants, to the longest recovery time of 15 months for one participant. CMC subjects with a mean age at recruitment of 55.6 ± 10.44 years, presented common cold symptoms or no symptoms during infection, resolved the disease at home within the year before sample collection, and did not report any symptoms at the moment of sample collection (**Table 1**).

**Table 1:** Characteristics of the groups included in the study.

	CSC	CMC	Control
N	18	14	9
Mean age (years)	54.3 ± 8.84	55.6 ± 10.44	53.3 ± 8.53
Female	6 (33.3%)	6 (42.8%)	5 (55.5%)
Ethnic origin	38% LA 5.5% ENS 5.5%M 51%ES	21% LA 79% ES	100% ES
Time from ICU			
4-5 months	2		
10-12 months	13		
13-15 months	3		

LA, Latin Americans; ENS, European non Spaniards; M, Moroccan; ES, European Spaniards. CSC, convalescent of severe COVID-19; CMC, convalescent of mild COVID-19.

Around half of the CSC subjects had a non-Caucasian ethnic origin, with a preponderance of the Andine region nationalities (Peru and Ecuador). During recruitment, CSC and CMC individuals were matched by ethnic origin when possible, but part of the CSC group was left unmatched (**Supplementary Tables 1, 2**). Ethnicity was the main difference between the matched and unmatched CSC participants, so divided the CSC group in participants of Spanish (Spanish-CSC) and Latin American (LA-CSC) origin, compared both subgroups in every variable and grouped the Spanish and Latin American CSC subjects in those analyses where no statistically significant differences or trends were found. All CSC and CMC participants were recruited and analyzed between May-September 2021.

Nine controls were recruited in a second phase in November-December 2021, before the outbreak of the Omicron strain in Spain, and were matched by sex and age with CMC and CSC subjects (**Supplementary Tables 1, 2**). Controls with a mean age at recruitment of 53.3 ± 8.53 years were all researchers and clinical personnel at Hospital Clínico San Carlos (Madrid, Spain). They passed two serological tests at the hospital with negative results during 2020, and were self-reportedly negative for COVID-19 from the beginning of the pandemics until recruitment. When recruited, all controls had received two doses of the vaccine and routine serology or memory T tests could not rule out a previous COVID-19 infection. Therefore, we selected participants who observed preventive measures such as social distancing and use of mask, that was mandatory in all public places in Spain at the moment of recruitment. Controls were interrogated about whether they had any suspicion of having passed COVID-19, if they had had any mild respiratory infection or had been in contact with a COVID-19 positive person since their last serologic test. All recruited controls answered “no” to the three questions.

Concerning sex, the differences in the proportion of males and females in controls, CMC and CSC reported in **Table 1** were not statistically significant when compared in contingency tables with Chi-square tests and the CSC groups stratified by ethnic origin had similar proportions of male and female participants (3 out of 9 in the Spanish-CSC and 3 out of 7 in the LA-CSC, **Supplementary Table 1**).

The mean age of the CSC group was similar to CMC and controls (**Table 1**), but within the CSC group, the LA-CSC participants were younger than their Spanish-CSC counterparts (mean age 48.3 y. o. vs. 58.3 y. o.). To identify if there could be an effect of age, we performed bivariate correlations between age and the analyzed variables in each group (controls, CMC and CSC) in the statistically significant results and we did not observe any influence or significant correlation. Therefore, age did not exert an effect in our sample on the variables studied.

All participants were unvaccinated or had one dose of the vaccine and controls had received two doses of the vaccine at the time of sample collection. In all cases, sample collection was performed at least three weeks after the administration of any vaccine. All participants signed a written informed consent and the study was approved by the Ethics Committee of Hospital Clínico San Carlos.

### 2.2 Flow cytometry

All participants donated 10 ml of peripheral blood anticoagulated with EDTA, and samples were processed within three hours after extraction. Briefly, 150 μl of peripheral blood was incubated with CD56-Pacific blue (362520), CD16-BrilliantViolet510 (302048), CD19-PercPCy5.5 (302230), NKG2A-APC fyre (375116), NKG2C-PE (375004), NKG2D-FITC (320820) and NKp44-APC (325110) from Biolegend (San Diego, CA, USA) and CD3-ECD (A07748) from Beckman Coulter (Brea, CA, USA), according to manufacturer’s protocols. After monoclonal incubation, red blood cells were lysed with BD FACS lysing solution (349202, Becton Dickinson, Franklin Lakes, NJ, USA). Samples were washed and suspended in 300 μl phosphate saline buffer, and acquired in a CytoFLEX flow cytometer (Beckman Coulter, Brea, CA). For every variable, all files were collected and data was analyzed at the same time with Kaluza v2.1 (Beckman Coulter, Brea, CA) by a blinded operator. NK^bright^, NK^dim^ and NKT populations were gated by expression of CD56, CD16 and CD3. Subpopulations expressing NKG2A, NKG2C and NKG2D were selected, and NKp44 expression was analyzed in every subpopulation with two different gating strategies to confirm the differences. Gating strategies are detailed in **Supplementary File**.

### 2.3 IL-6 measurement

A general biochemistry was performed in serum extracted from CSC and CMC subjects by the Central Laboratory services at Hospital Clínico San Carlos. IL-6 was included in the analysis as an inflammatory marker commonly used during diagnosis of severe COVID-19, and was analyzed by electroquimioluminiscence assay (Elecsys IL-6, cat. n. 05109442190, Roche Diagnostics) in a Cobas analyzer (Roche Diagnostics) following standard clinical techniques. Normality levels (0.1-7 pg/ml) were fixed by the Laboratory services, according to the provider’s instructions.

### 2.4 Statistical analyses

Sample size was calculated to have 80% statistical power to detect a minimum difference of 5 units with common standard deviation of 4 between CMC and CSC patients. Control sample size was calculated to reach 80% statistical power based on the registered differences between CMC and CSC groups. Data were collected and normality assessed in all groups by Kolmogorov-Smirnov test. Outliers in every group were detected with Grubb’s test in GraphPad online tool (https://www.graphpad.com/quickcalcs/Grubbs1.cfm). Differences in the mean distribution of all variables were analyzed by ANOVA and t-Student tests. Statistical analyses and graphical representations were performed with SPSS v15.0.1 (Chicago, Illinois, USA) and GraphPad Prism V8.01.

## 3 Results

### 3.1 Convalescent individuals of severe COVID-19 have a lower CD56^bright^ NK/CD56dim NK ratio

**Figure 1:**
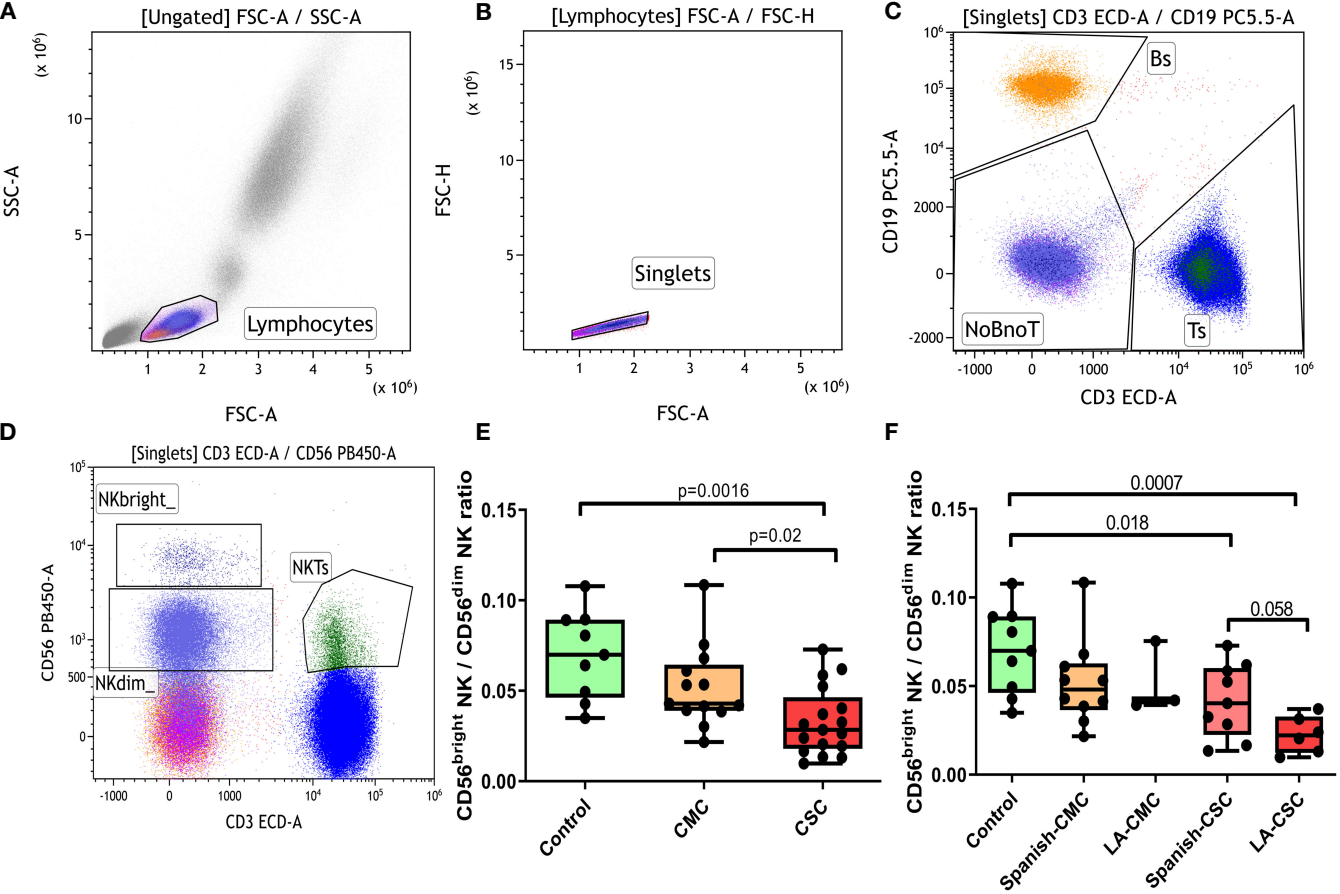
Analysis of the CD56^bright^ NK/CD56^dim^ NK ratio. **(A-D)** Overall gating strategy for the selection of T, B and NK populations: **(A)** Selection of lymphocytes by forward and side scatter. **(B)** Selection of single events. **(C)** Gating for T and B populations based on CD3 or CD19 positivity. **(D)** Selection of T, NK^bright^, NK^dim^ and NKT populations based on the expression patterns of CD3 and CD56 antigens. **(E)** CD56^bright^ NK/CD56^dim^ NK ratio in controls, CMC and CSC. **(F)** CD56^bright^ NK/CD56^dim^ NK ratio stratified by ethnic origin of the participants. CMC/CSC: convalescent of mild/severe COVID-19 individuals. LA-CSC/CMC: Latin-American convalescent of severe/mild COVID-19. Boxes represent the 25 and 75% quartiles, lines represent the median of the distribution, and whiskers are the maximum and minimum values of the distribution.

Overall T, B, NK and NKT cellularity was assessed in controls, CMC and CSC participants, and no significant differences among groups were detected. However, trends for a lower percentage of CD56^bright^ NK and a higher CD56^dim^ NK were found in CMC and CSC groups compared to controls (gating strategy shown in **Figures 1A–D**). When the ratio CD56^bright^NK/CD56^dim^ NK was calculated, a statistically significant decrease was evidenced in the CSC group compared to controls (**Figure 1E**). The CMC group showed significantly higher ratio than the CSC one, and a mild decrease that did not reach statistical significance when compared to controls.

The CD56^bright^ NK/CD56^dim^ NK ratio was also analyzed after stratifying groups according to ethnic origin: Spaniards of Caucasian descent (Spanish-CSC) and Latin Americans (LA-CSC, **Figure 1F**). Interestingly, the LA-CSC group showed the lowest CD56^bright^ NK/CD56^dim^NK ratio of all groups and a nearly significant trend to a lower ratio compared to the Spanish-CSC (p=0.058). No difference according to ethnicity was detected in the CMC category.

### 3.2 Convalescent individuals of severe COVID-19 have higher NKp44 expression in CD56^bright^ NK subpopulations

**Figure 2:**
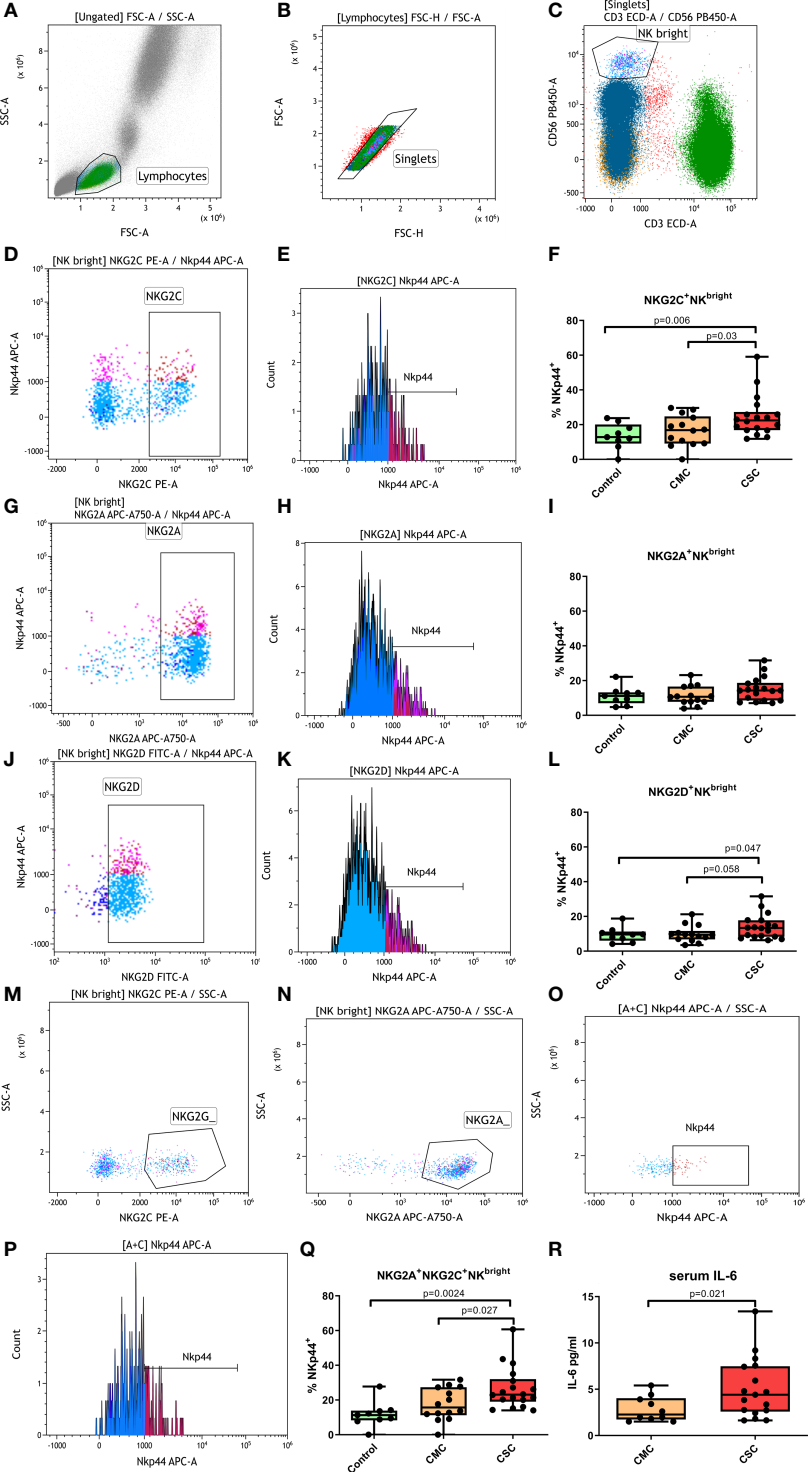
Percentage of NKp44^+^ cells in CD56^bright^ NK subpopulations. **(A-C)** Overall gating strategy: **(A)** Selection of lymphocytes by forward and side scatter. **(B)** Selection of single events. **(C)** Selection of CD56^bright^ NK cells. **(D)** Selection of NKG2C^+^ CD56^bright^ NK cells. **(E)** Selection of NKp44^+^ cells within the NKG2C^+^CD56^bright^ NK population. **(F)** Percentage of NKp44^+^ NKG2C^+^ CD56^bright^ NK cells in controls, CMC and CSC. **(G)** Selection of NKG2A^+^CD56^bright^ NK cells. **(H)** Selection of NKp44^+^ cells within the NKG2A^+^CD56^bright^ NK population. **(I)** Percentage of NKp44^+^ NKG2A^+^CD56^bright^ NK cells in controls, CMC and CSC. **(J)** Selection of NKG2D^+^CD56^bright^ NK cells. **(K)** Selection of NKp44^+^ cells within the NKG2D^+^ CD56^bright^ NK population. **(L)** Percentage of NKp44^+^NKG2C^+^CD56^bright^ NK cells in controls, CMC and CSC. **(M, N)** Selection of NKG2A^+^NKG2C^+^CD56^bright^ NK cells by a boolean gate that included all events in the CD56^bright^ window that were positive for NKG2C^+^ **(M)** and NKG2A^+^ **(N)**. **(O, P)**: Percentage of NKp44^+^ cells in the NKG2A^+^NKG2C^+^CD56^bright^ NK population. The NKp44^+^ population was determined in a histogram plot **(P)** and confirmed in a dot plot **(O)**. **(Q)** Percentage of NKp44^+^ NKG2A^+^NKG2C^+^CD56^bright^ NK cells in controls, CMC and CSC. **(R)** Levels of serum IL-6 in CMC and CSC. CMC/CSC: convalescent of mild/severe COVID-19 individuals. Boxes represent the 25 and 75% quartiles, lines represent the median of the distribution, and whiskers are the maximum and minimum values of the distribution.

We analyzed the populations with the inhibitory receptor NKG2A and the activating receptors NKG2C and NKG2D in CD56^dim^ NK, CD56^bright^NK and NKT cells. Subpopulations expressing these receptors were altered in patients during active COVID-19 (3). Nonetheless, months after disease resolution, our participants did not present significant changes in the percentages of NK cells that expressed these receptors. When the distribution of the activating receptor NKp44 was analyzed (gating strategy shown in **Figure 2**), we found interesting differences in the CD56^bright^NK subpopulation. The CSC group had significantly higher levels of NKp44 in the NKG2C^+^CD56^bright^NK subpopulation compared to CMC and control groups (**Figure 2F**). These differences were also observed in the combined NKG2A^+^NKG2C^+^CD56^bright^NK population (**Figure 2Q**), and a smaller but still significant NKp44 increase was reproduced in the NKG2D^+^CD56^bright^NK subpopulation (**Figure 2L**). The NKG2A^+^CD56^bright^NK population showed no statistically significant differences among these three groups (**Figure 2I**). No differences according to ethnic origin of the participants were detected (**Supplementary Figures 1A, B**).

### 3.3 Serum IL-6 is elevated months after severe COVID-19

Markers of inflammation such as IL-6 and C reactive protein, as well as a biochemistry analysis, were performed to CMC and CSC subjects at the moment of blood sample collection. Higher IL-6 levels were observed in the CSC compared to the CMC group (**Figure 2R**). Moreover, a trend for a higher rate of IL-6 positivity in the CSC was found compared to the CMC group. While in the CSC group five out of 18 subjects presented higher IL-6 levels than the normality range months after disease resolution, the 10 individuals in the CMC group with IL-6 data moved within the normality range. This difference was not statistically significant (p=0.13, OR=8.5 [0.42-172.7]), probably due to low statistical power. The increased IL-6 levels were not correlated with other variables in the biochemistry analysis, such as cholesterol or glucose levels and differences according to ethnic origin of the participants were not detected (**Supplementary Figure 1C**).

### 3.4 B cells show a trend to a lower surface expression of CD19 in convalescent of severe COVID-19 individuals

**Figure 3:**
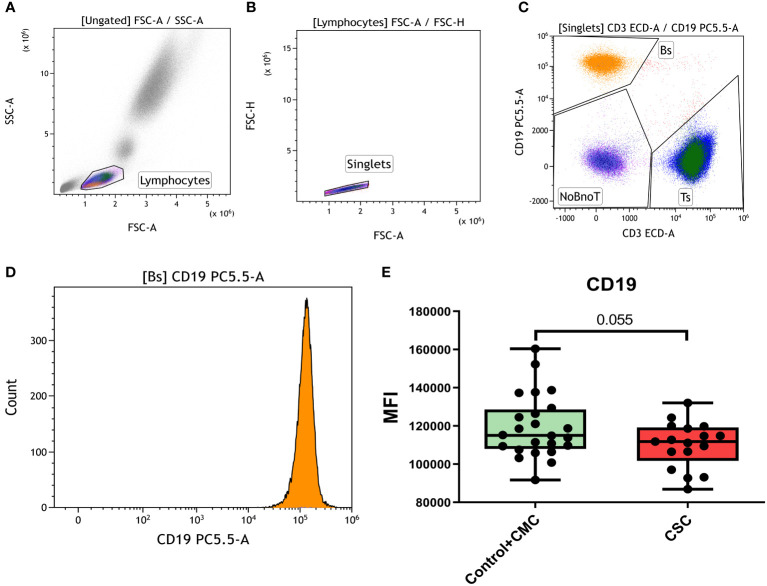
Expression of CD19 in B lymphocytes. **(A)** Selection of lymphocytes by forward and side scatter. **(B)** Selection of single events. **(C)** Determination of general lymphocyte populations by CD3 and CD19 and gating of the CD19^+^ population. **(D)** Median fluorescence intensity (MFI) of CD19 in B cells. **(E)** Differences in CD19 expression measured as MFI in the Control+CMC group and in CSC participants. CMC/CSC: convalescent of mild/severe COVID-19 individuals. Boxes represent the 25 and 75% quartiles, lines represent the median of the distribution, and whiskers are the maximum and minimum values of the distribution.

A lower surface expression of CD19, among other alterations in the B cell compartment, was reported in CSC subjects at three months after recovery (15). In our population (gating strategy shown in **Figures 3A–D**), the CD19 surface levels in controls and the CMC individuals were similar, but the CSC group showed a trend to a lower expression. Considering that the CD19 expression levels in the control and CMC groups were similar, CSC subjects were compared with the combined control and CMC group to increase the statistical power, and a nearly significant trend was then evidenced (p=0.055, **Figure 3**). When stratified by ethnic origin of the participants, no relevant differences were detected between groups (**Supplementary Figure 1D**).

### 3.5 Individuals recovered from severe COVID-19 have lower levels of NKG2A^+^CD3^+^CD56^-^ T cells

**Figure 4:**
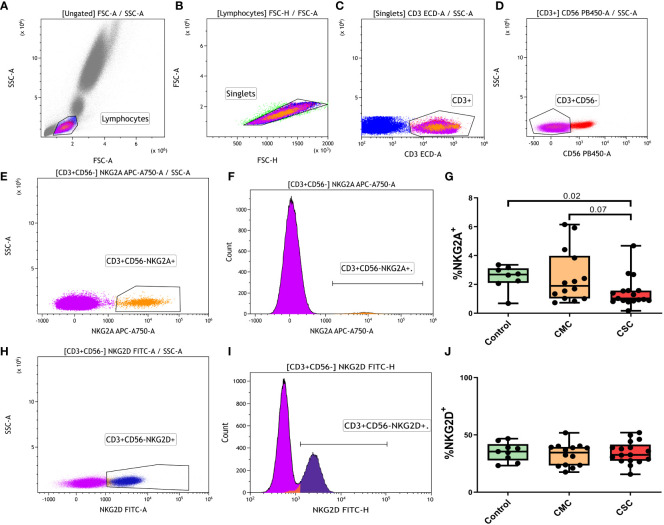
Percentage of NKG2A^+^ and NKG2D^+^ cells in the CD3^+^CD56^-^ T population. **(A-D)** Gating strategy for the selection of the CD3^+^CD56^-^ T population: **(A)** Gating of lymphocytes by forward and side scatter. **(B)** Selection of single events. **(C)** Selection of CD3^+^ events. **(D)** Selection of the CD3^+^CD56^-^ events in the CD3^+^ lymphocyte gate. **(E, F)** Selection of the NKG2A^+^ CD3^+^CD56^-^ T cells by dot plot **(E)** and histogram plot **(F)**. **(G)** Differences in the percentage of NKG2A^+^CD3^+^CD56^-^ T cells in controls, CMC and CSC participants. **(H, I)** Selection of the NKG2D^+^ CD3^+^CD56^-^ T cells by dot plot **(H)** and histogram plot **(I)**. **(J)** Differences in the percentage of NKG2D^+^CD3^+^CD56^-^ T cells in controls, CMC and CSC participants. CMC/CSC: convalescent of mild/severe COVID-19 individuals. Boxes represent the 25 and 75% quartiles, lines represent the median of the distribution, and whiskers are the maximum and minimum values of the distribution.

Previous studies highlighted the roles of NKG2A and NKG2D receptors in CD8^+^T cells during active COVID-19 (3, 20). Our analysis was designed to study NK cells and, therefore, we could not gate the CD8^+^T population, so we analyzed the expression of NKG2A and NKG2D in CD3^+^CD56^-^ T cells (**Figures 4A–D**). The CSC group showed lower levels of NKG2A^+^CD3^+^CD56^-^T cells than the control and CMC groups (**Figures 4E–G**). No significant differences in the NKG2D marker were found between control, CMC and CSC groups (**Figures 4H–J**) and stratification by ethnic origin of the participants did not provide relevant results (**Supplementary Figure 1E**).

## 4 Discussion

NK cells are key players in the antiviral response due to their cytotoxic, cytolytic and cytokine-producing activities. Certain subpopulations of NK cells may react differently to the environment depending on the signals perceived from the inflammatory milieu (4, 10, 21) or the nature of interactions with target cells (10), so they can be friends or foes depending on the stimuli received. Several studies reported alterations in the NK cell compartment during active severe COVID-19. These alterations included lower numbers of NK cells (3) and higher expression of NKG2C (3) and of the activating marker NKp44 (8, 9). Interestingly, our CSC group shows higher levels of NKp44 in the NKG2C^+^ CD56^bright^ NK subpopulation, months after disease clearance.

NKG2C^+^NK cells are considered a memory subset in the NK cell population for their ability to proliferate in response to cytomegalovirus antigens and other viral infections (6, 7). In our study, after several months of convalescence, the number of NKG2C^+^NK cells in the CSC group is similar to those in CMC and control groups. However, even though this population seems to recover normality, a higher expression of the activating receptor NKp44 was detected in the NKG2C^+^, NKG2A^+^C^+^ and NKG2D^+^CD56^bright^ NK subpopulations in CSC subjects, indicative of the still compromised activity of these subpopulations. NKp44 is traditionally considered an activating receptor and transduces signals through an ITAM (immunoreceptor tyrosine-based activating motif) adaptive protein. Nonetheless, it also presents splicing variants which function as ITIM (immunoreceptor tyrosine-based inhibitory motif) sequences (10, 22), and therefore its actions may be different depending on the engaged ligands. The most significant rises in levels of NKp44 expression are detected in NKG2C^+^ and NKG2A^+^C^+^CD56^bright^ NK populations. Milder effects observed in the NKG2D^+^NK^bright^ population are probably a reflection of the differences observed in the NKG2C^+^ and NKG2A^+^C^+^CD56^bright^ NK populations, since the NKG2D^+^ receptor is expressed by overall 90% of the NK cells. As mentioned before, NKG2C^+^ NK cells are responsive to viral antigens, especially cytomegalovirus. The combined NKG2A^+^C^+^ population originates from NKG2C^+^ NK cells that express the inhibiting receptor NKG2A^+^ as a way to downregulate the antiviral response (23). The higher expression levels of NKp44 in NKG2C^+^CD56^bright^ NK cells could be a remnant of a past exacerbated activation or a sign of immune exhaustion in these particular subsets, as they are only detected in the CSC group. However, its biological significance in these individuals is uncertain.

We observe an imbalance in the proportions of CD56^bright^ NK and CD56^dim^ NK cells months after hospital discharge of the CSC subjects, with a lower level of CD56^bright^ NK cells, and, therefore, a lower CD56^bright^ NK/CD56^dim^ NK ratio. Varchetta et al. (24) found lower levels of CD56^bright^ NK and a parallel increase of CD56^dim^ NK cells during active COVID-19 in severe hospitalized patients compared to healthy controls, a disequilibrium that was more prominent in those patients who died. Bergantini et al. (8) found the same imbalance in hospitalized COVID-19 patients, a study cohort that included patients under low flow and high flow oxygen therapy, and patients in ICU with mechanical ventilation. This imbalance may have an impact in the regulatory functions of the NK cell population. Alterations in the CD56^bright^ NK/CD56^dim^ NK ratio have been previously described in autoimmune diseases such as primary Sjögren disease (25), were CD56^bright^ NK cells are increased, or systemic lupus erythematosus, with CD56^bright^ NK cells in lower numbers than in healthy controls (26). Studies on the effect of treatments for multiple sclerosis in the NK cell compartment reported that several disease modifying treatments increased the proportion of CD56^bright^ NK cells and the CD56^bright^ NK/CD56^dim^ NK ratio (27). Considering the cytokine-producing potential of the CD56^bright^ NK compartment, this increase was interpreted as positive for their immunoregulatory potential. In view of the previous COVID-19 studies that have identified lower CD56^bright^ NK/CD56^dim^ NK ratios in severe patients undergoing disease, the imbalance that we detect could be a vestige of perturbations generated during active disease, which persist months after hospital discharge.

Interestingly, the CSC group also shows lower levels of the NKG2A^+^ marker in the CD3^+^CD56^-^T population. NKG2A has been reported to increase in correlation with CD8^+^T and NK cell exhaustion during COVID-19 progression, with decreasing levels during convalescence (28). The lower levels of NKG2A detected in the CSC group, together with the other observed alterations, suggest a delayed recovery of the immune system in the CSC group that has already taken place in the CMC group. Our study included CSC participants with an average of eleven months after ICU admission, being one of the studies in the literature with a longer convalescent period, but we still detect perturbations in the immune system.

The CD56^bright^ NK/CD56^dim^ NK imbalance shows a trend to be stronger in those individuals in our cohort with Latin-American origin compared to those Spaniards with Caucasian origin. Several studies in public hospitals in Spain detected a higher proportion of ICU admissions among patients with Latin American origin, even after adjustment for socioeconomic variables such as residence area, population density in that area and *per capita* income average (29–33). This altered CD56^bright^ NK/CD56^dim^ NK ratio in convalescent patients of Latin American origin could be a consequence of the higher severity observed in several studies in Spain. However, this is the only effect related to ethnicity detected in our analyses, and the results must be taken cautiously owing to the sample size. Although some authors are critical with the reported ethnic differences in severity, since many of the studies do not adjust by socioeconomic variables (exposure conditioned by type of job, commuting in public transport, shared household, educational level…) (34), the abovementioned Spanish studies adjusted their analyses by age, sex and basic socioeconomic variables. Most agreed in a higher ICU admission with comparable mortality rate between Latin American and Spanish patients, which would support a higher severity in the Latin American population. The lower CD56^bright^ NK/CD56^dim^ NK ratio detected in our population would correlate with the higher severity and ICU admission of Latin Americans observed in several regions of Spain.

As a limitation of our study, we should indicate that controls were already vaccinated when they enroled and we did not perform serological tests to rule out previous infection. Control samples were gathered before the outbreak of the highly spreadable Omicron strain in Spain in mid-December 2021. Before that, the incidence of COVID-19 among the research personnel of our facility was minimal. Spain had very restrictive measures concerning COVID-19 prevention during 2020 and 2021, with a mandatory use of mask in all public spaces. It could be possible that some controls had experimented asymptomatic COVID-19 before recruitment, but it is unlikely considering how the pandemics affected our surroundings. Moreover, controls were recruited specially to check differences with CMC participants. If we analyze the control data, most variables are homogeneous, and we do not detect differences or statistical trends between CMCs and controls. If any effect of mild COVID-19 infection on the immune system would have existed, and we had a small control group contaminated with convalescent mild COVID-19 individuals, we would expect the data to reflect that contamination with a higher dispersion.

Vaccination status was not included in the analyses because we had incomplete registers for the CMC and CSC patients. However, with a fully vaccinated control group and half of the CMC group with one or two doses, we see no evidence of the vaccination status affecting the variables that we report, as expected since the vaccine would mimic a mild or asymptomatic infection. We do not appreciate any evidence of mild or asymptomatic infection having an effect on the variables studied and, to our knowledge, there is no report in the literature of mild COVID-19 having long-term effects on the immune system.

Another potential limitation would be the absence of a viability dye in the cytometry panel to exclude dead or apoptotic cells from the analysis window. Dead cells have a higher autofluorescence and tend to bind antibodies unspecifically, so they could distort cytometry readings. Fresh blood samples stained with surface markers have a very low amount of dead or apoptotic cells, the blood populations are clear and easy to gate and it is not uncommon to remove the viability dye. We used an unmarked sample to check autofluorescence and the results reflected a negligible amount of autofluorescence in the analysis gates (see **Supplementary File**), specially in those populations were we see statistically significant results. False positives related to the presence of dead cells in the analysis window affect specially low frequency populations. We analyzed many low-frequency populations in controls, CMC and CSC, and we only observed increases in specific cell populations in CSC participants. The presence of dead autofluorescent cells would affect the results more randomly and would probably produce a higher dispersion of the data.

In summary, we find alterations in the NK, T and B cell compartments that persist close to a year after hospital discharge in subjects who suffered severe COVID-19. These results are in line with previous reports of alterations in the immune cell compartment that are still detected several months after resolution of the COVID-19 symptoms.

## Data availability statement

The original contributions presented in the study are included in the article/**Supplementary Material**. Further inquiries can be directed to the corresponding author/s.

## Ethics statement

The studies involving human participants were reviewed and approved by Ethics comittee of Hospital Clínico San Carlos. The patients/participants provided their written informed consent to participate in this study.

## Author contributions

EU and LE-P designed the study, analyzed the data and wrote the manuscript. JA-Z, MC-R, AG-J, ARL-P, TF, CR-A and EA collected the samples and clinical data, analyzed the data and performed research. All authors contributed to the article and approved the submitted version.
