# Supplementary material for: Alterations in the immune system persist after one year of convalescence in severe COVID-19 patients
Source: Front Immunol. 2023 Feb 13;14:1127352. doi: 10.3389/fimmu.2023.1127352 (PMC9969554; doi:10.3389/fimmu.2023.1127352)
Supplement: Supplementary file 1 [file DataSheet_1.zip › Supplementary figure 1.docx]

**Supplementary figure 1.-Stratified analysis by ethnic origin of the participants.**


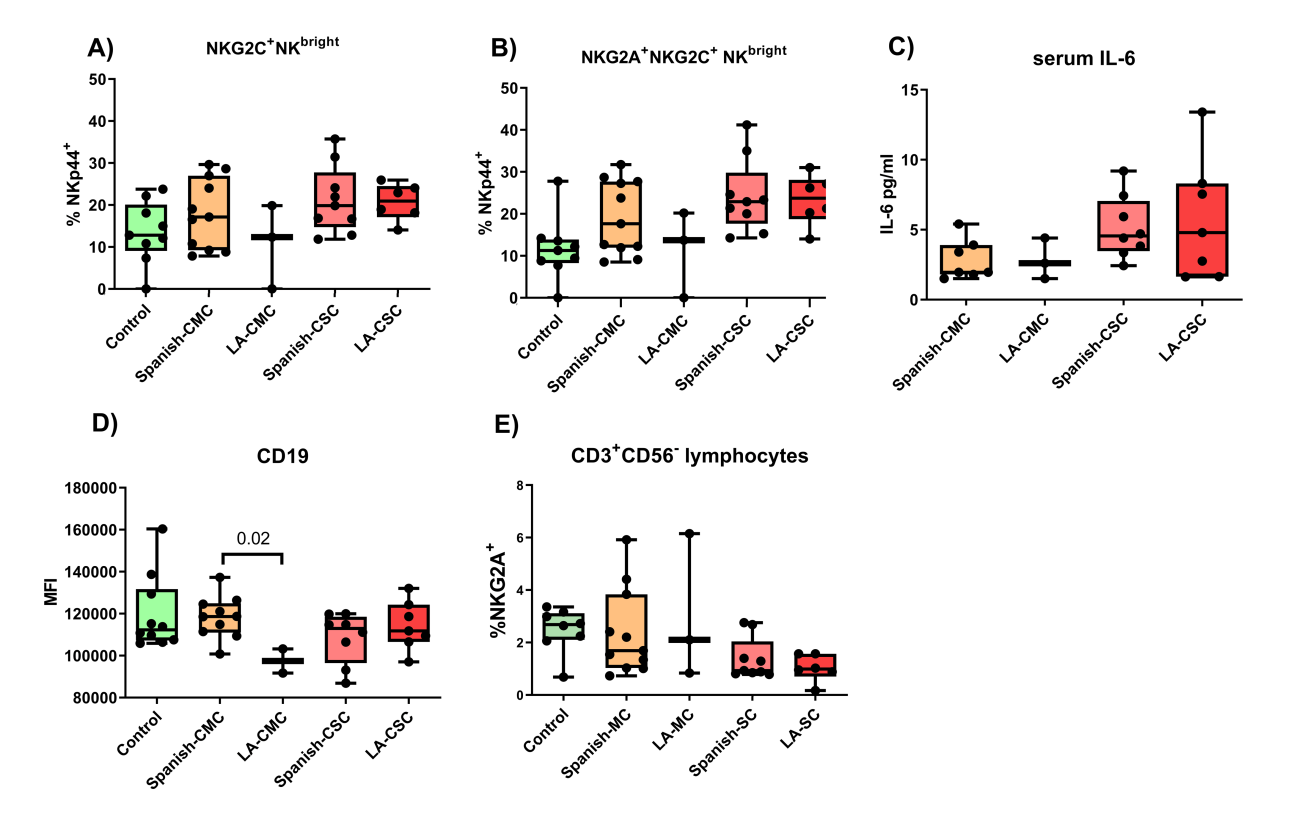


Statistical results are only included when differences between convalescent of severe COVID-19 (CSC) ethnic groups are registered. A) Percentages of NKp44^+^NKG2C^+^CD56^bright^NK cells. B) Percentages of NKp44^+^NKG2A^+^NKG2C^+^CD56^bright^NK cells. C) Expression levels of CD19 in B cells, measured as median fluorescence intensity (MFI). D) Serum IL-6 levels in CMC and CSC participants. E) Percentage of NKG2A^+^ in T cells (CD3^+^CD56^-^). CMC/CSC: convalescent of mild/severe COVID-19 subject. Boxes represent the 25 and 75% quartiles, lines represent the median of the distribution, and whiskers the maximum and minimum values of the distribution. **Note:** a statistical difference between Spanish-CMC and LA-CMC was detected in CD19 expression. However, these data must be taken with extreme caution since the LA-CMC group had only two participants, and there is no statistically significant difference between this group, and controls or the CSC groups. The registered difference is probably due to chance.
