# Supplementary material for: Alterations in the immune system persist after one year of convalescence in severe COVID-19 patients
Source: Front Immunol. 2023 Feb 13;14:1127352. doi: 10.3389/fimmu.2023.1127352 (PMC9969554; doi:10.3389/fimmu.2023.1127352)
Supplement: Supplementary file 1 [file DataSheet_1.zip › Supplementary file.pdf]

Supplementary file 1: gating strategy for NK^{bright}, NK^{dim}, NKT and CD19 expression

See "Note" and page 3 analysis for NK^{bright}

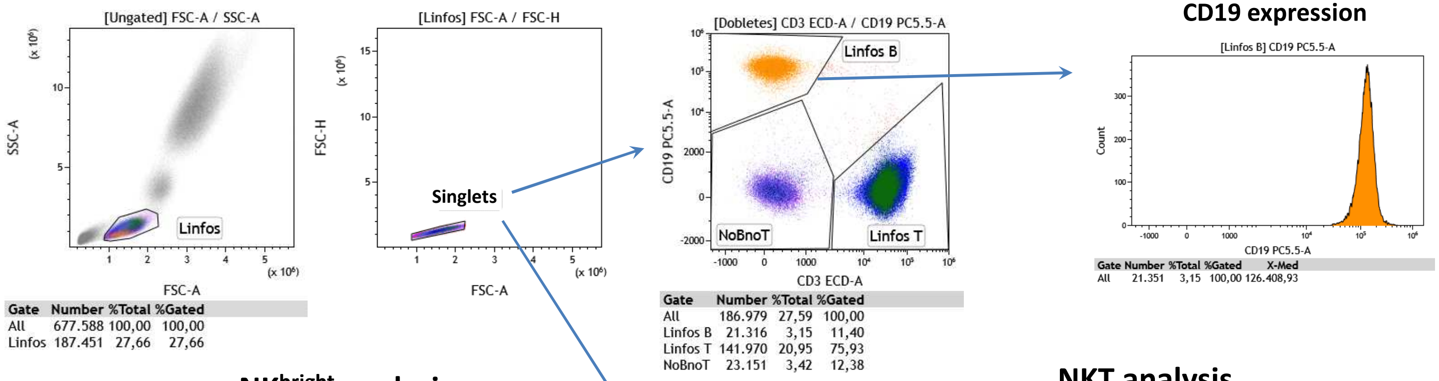

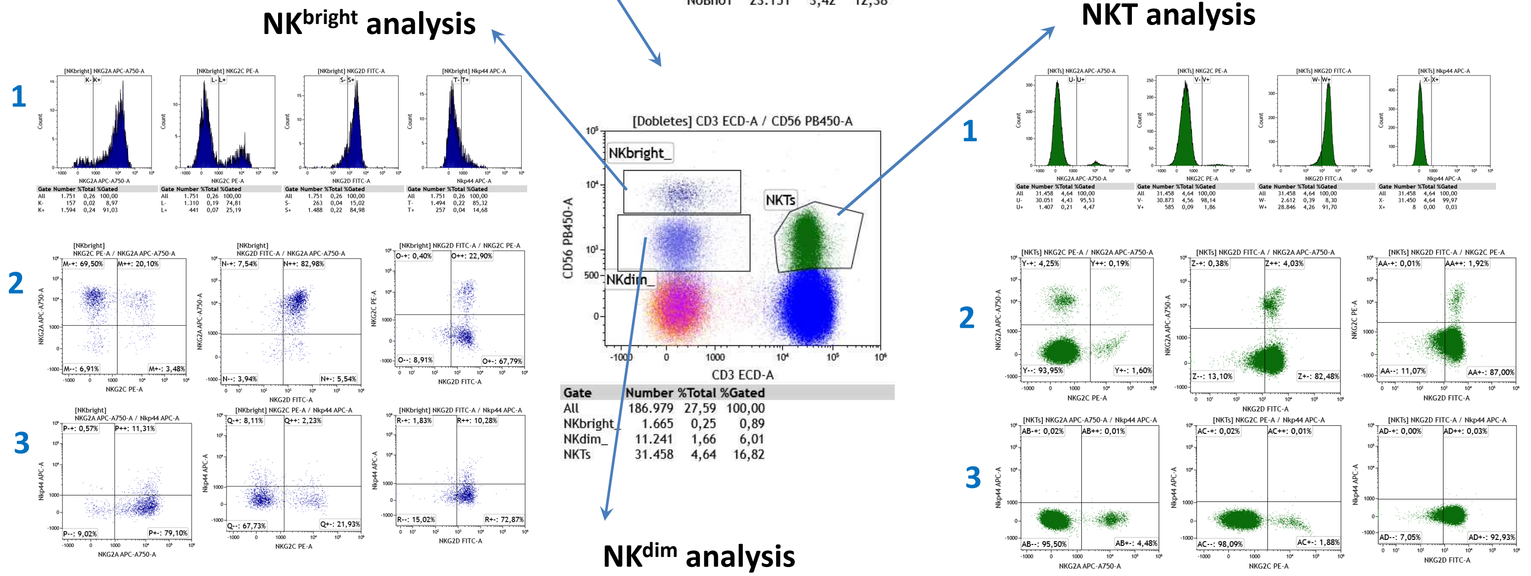

Analysis of the NK^{bright}, NK^{dim} and NKT populations

Three analyses were performed in every population:

1. Percentage of cells that express NKG2A, NKG2C, NKG2D or NKp44.
2. Percentage of cells that express combinations of NKG2A, NKG2C and NKG2D
3. Expression of NKp44 in NKG2A^{+}, NKG2C^{+} or NKG2D^{+} populations

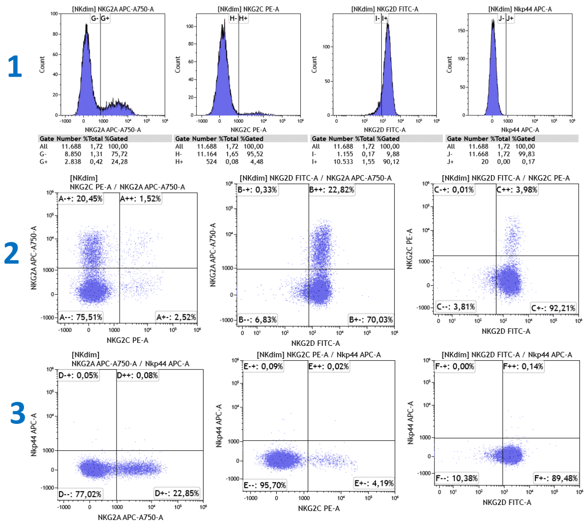

NOTE: A specific gating strategy for the expression of NKp44 in NK^{bright} was designed (see page 3) after differences in this analysis were detected.

The analysis presented in the paper for NK^{bright} populations is the one detailed in the next page. Both strategies rendered the same differences.

Supplementary file 1: autofluorescence control for NK^{bright}, NK^{dim}, NKT and CD19 expression

Autofluorescence determination obtained with a blood sample unstained and processed as the stained blood sample

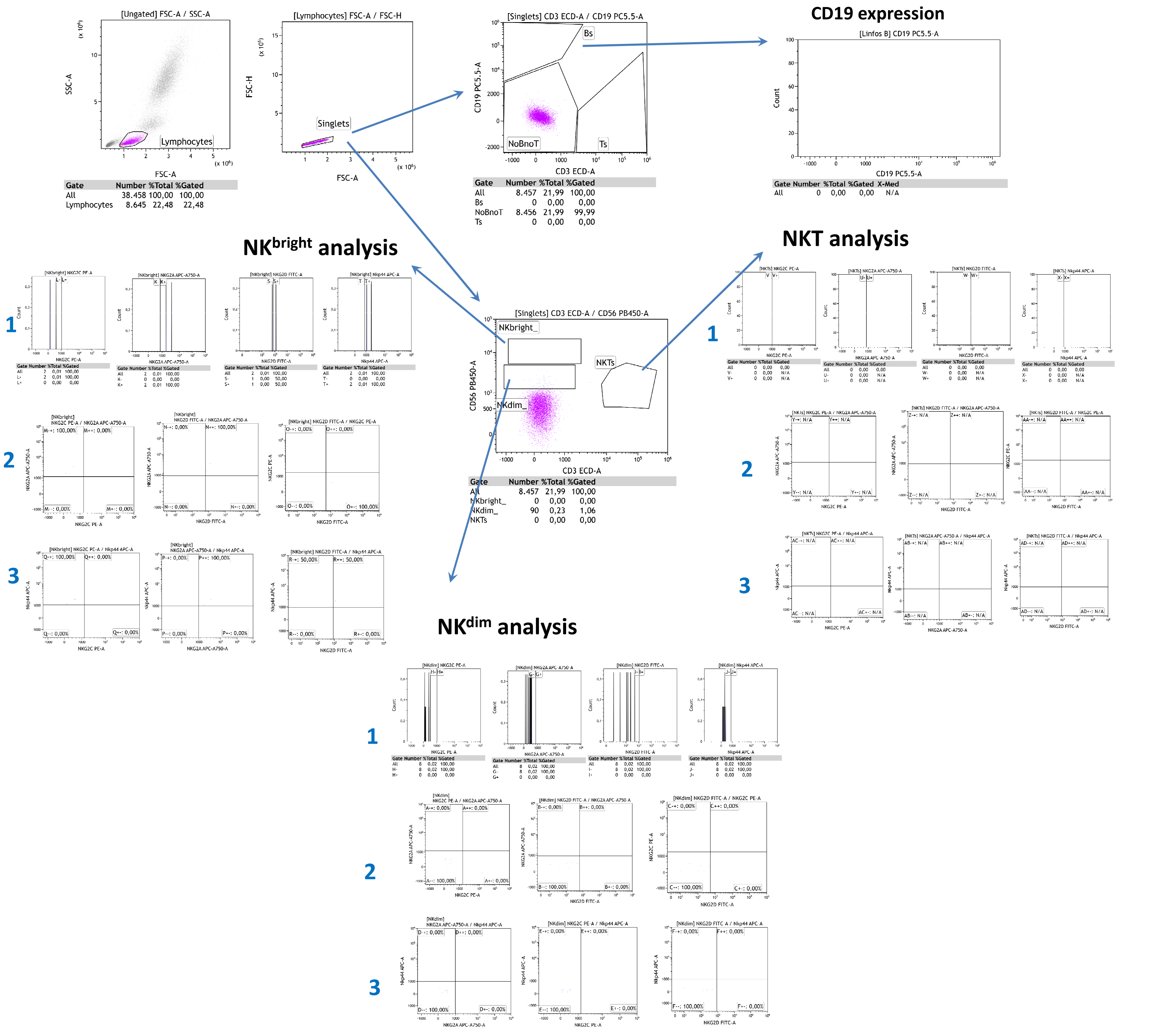

Supplementary file 1: gating strategy for the expression of NKp44 in NK^{bright} subpopulations

Data presented in the paper

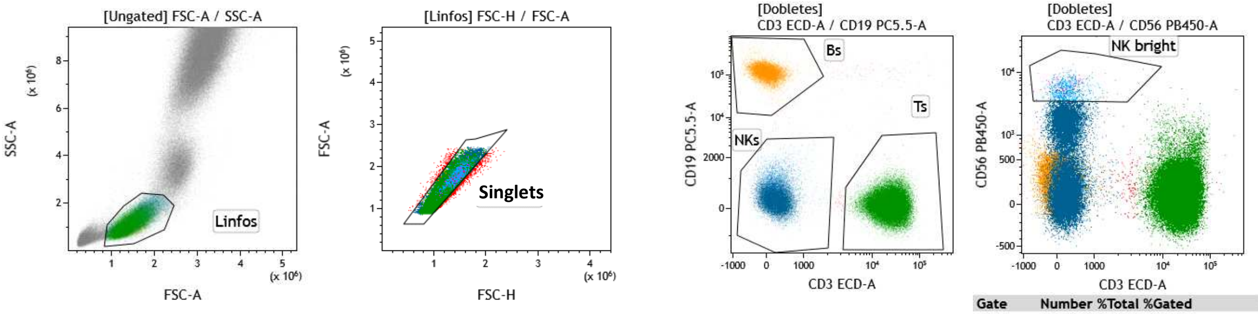

Gate	Number	%Total	%Gated
All	96.431	18,28	100,00
NK bright	825	0,16	0,86

Analysis of NKp44 in the NK^{bright} subpopulations:

Once the NK^{bright} population was gated, plots for NKG2C, NKG2A and NKG2D were created and positive populations selected (1). NKp44 intensity was represented in a histogram for each subpopulation and NKp44^{+} cells were gated in the histogram (2) and checked in the dot plot (1).

For the combined NKG2A^{+}NKG2C^{+} population (3), a boolean gate was created that combined the cells that were positive for both markers (gates G+H). Expression of NKp44 in this population was plotted in a dot plot and a histogram. NKp44^{+} cells were gated in the histogram and checked in the dot plot.

1

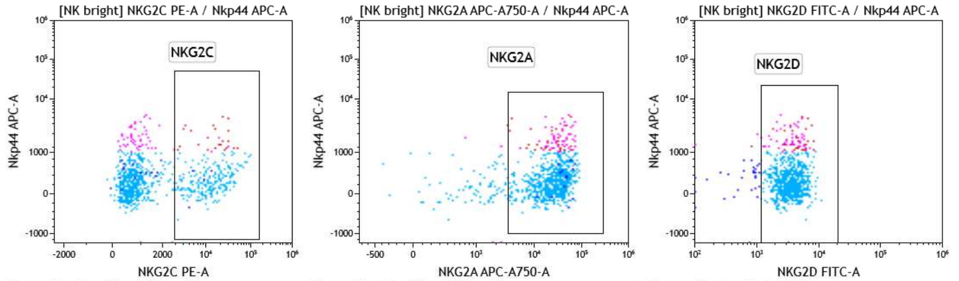

Gate	Number	%Total	%Gated
All	825	0,16	100,00
NKG2C	254	0,05	30,79

Gate	Number	%Total	%Gated
All	825	0,16	100,00
NKG2A	745	0,14	90,30

Gate	Number	%Total	%Gated
All	825	0,16	100,00
NKG2D	792	0,15	96,00

2

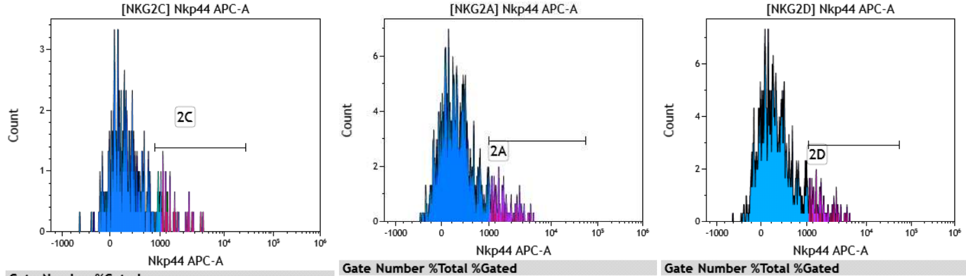

Gate	Number	%Gated
All	254	100,00
2C	38	14,96

Gate	Number	%Total	%Gated
All	745	0,14	100,00
2A	83	0,02	11,14

Gate	Number	%Total	%Gated
All	792	0,15	100,00
2D	77	0,01	9,72

3

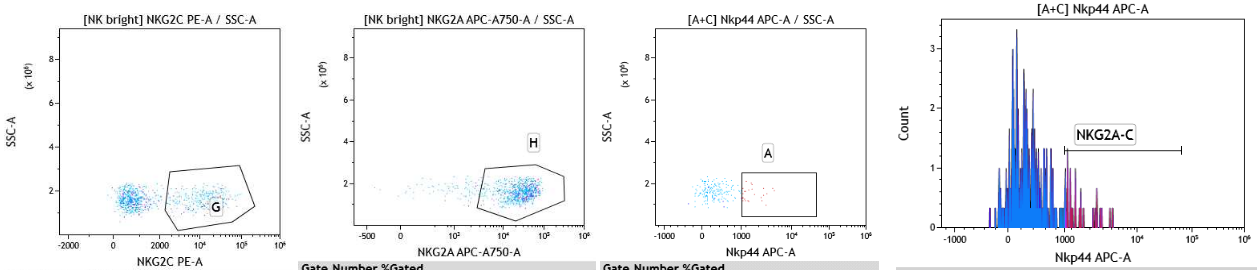

Gate	Number	%Gated
All	825	100,00
G	263	31,88

Gate	Number	%Gated
All	825	100,00
H	743	90,06

Gate	Number	%Gated
All	205	100,00
A	25	12,20

Gate	Number	%Total	%Gated
All	205	0,04	100,00
NKG2A-C	28	0,01	13,66

Supplementary file 1: autofluorescence control for the expression of NKp44 in NK^{bright} subpopulations

Autofluorescence determination obtained with a blood sample unstained and processed as the stained blood sample

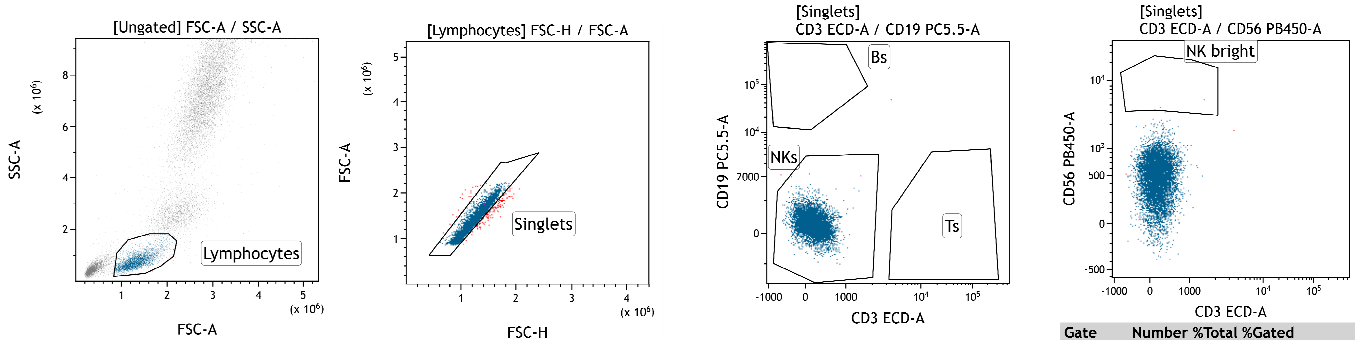

Gate	Number	%Total	%Gated
All	8.713	22,66	100,00
NK bright	3	0,01	0,03

1

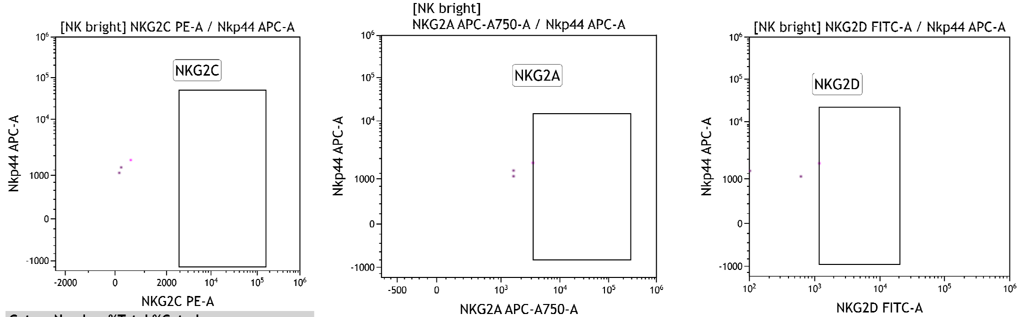

Gate	Number	%Total	%Gated
All	3	0,01	100,00
NKG2C	0	0,00	0,00

Gate	Number	%Total	%Gated
All	3	0,01	100,00
NKG2A	0	0,00	0,00

Gate	Number	%Total	%Gated
All	3	0,01	100,00
NKG2D	1	0,00	33,33

2

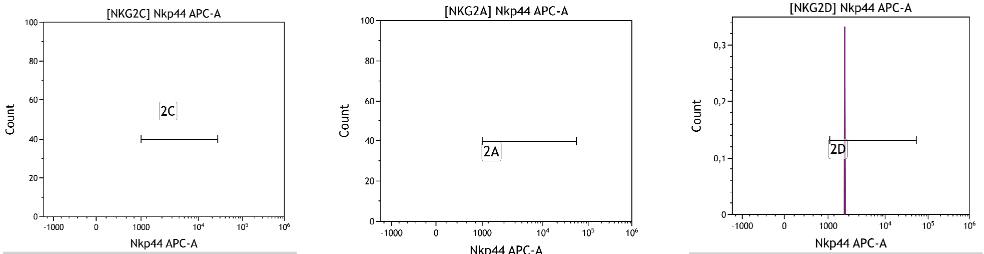

Gate	Number	%Gated
All	0	0,00
2C	0	N/A

Gate	Number	%Total	%Gated
All	0	0,00	0,00
2A	0	0,00	N/A

Gate	Number	%Total	%Gated
All	1	0,00	100,00
2D	1	0,00	100,00

3

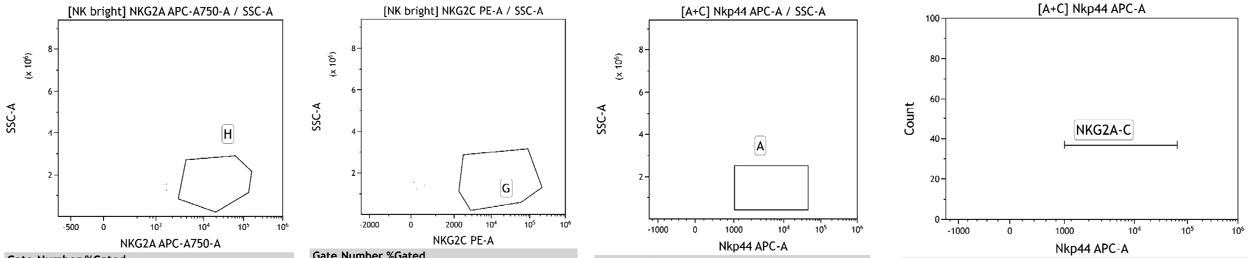

Gate	Number	%Gated
All	3	100,00
H	1	33,33

Gate	Number	%Gated
All	3	100,00
G	0	0,00

Gate	Number	%Gated
All	0	N/A
A	0	N/A

Gate	Number	%Total	%Gated
All	0	0,00	N/A
NKG2A-C	0	0,00	N/A

Supplementary file 1: gating strategy for NKG2A^{+}CD3^{+}CD56^{-} and NKG2D^{+}CD3^{+}CD56^{-} populations

Data presented in the paper

1. NKG2A^{+}CD3^{+}CD56^{-}

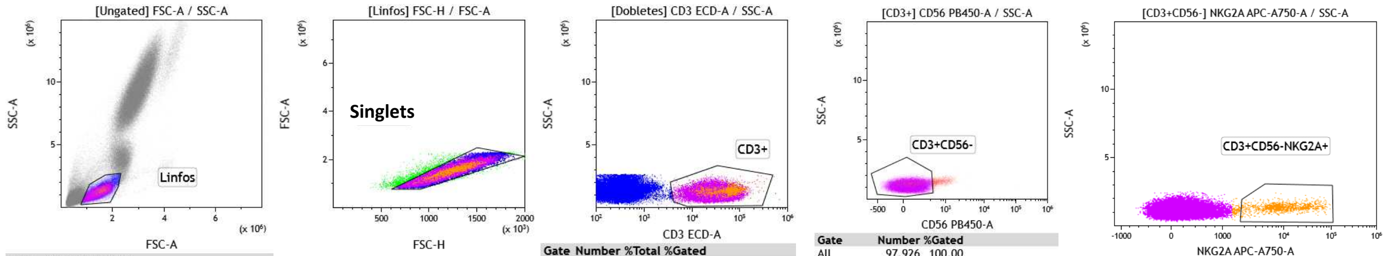

Gate	Number	%Total
All	653.065	100,00
Linfos	188.946	28,93

Gate	Number	%Total	%Gated
All	187.362	28,69	100,00
CD3+	97.926	14,99	52,27

Gate	Number	%Gated
All	97.926	100,00
CD3+CD56-	95.306	97,32

Gate	Number	%Total	%Gated
All	95.306	14,59	100,00
CD3+CD56-NKG2A+	1.229	0,19	1,29

2. NKG2D^{+}CD3^{+}CD56^{-}

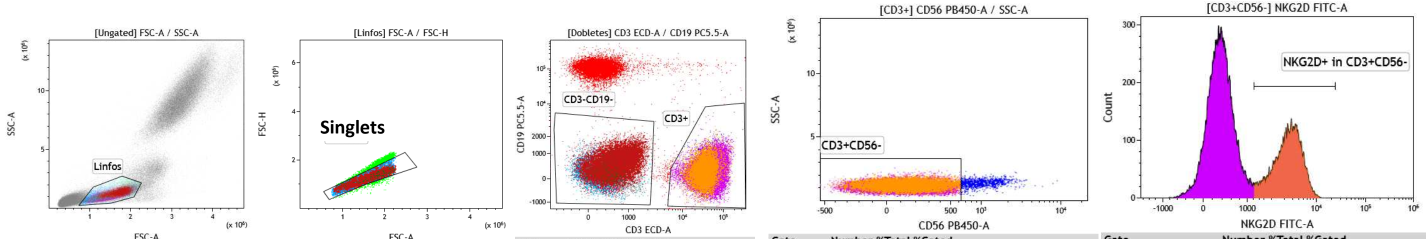

Gate	Number	%Total	%Gated
All	304.338	100,00	100,00
Linfos	101.552	33,37	33,37

Gate	Number	%Total	%Gated
All	99.354	32,65	100,00
CD3-CD19-	37.181	12,22	37,42
CD3+	48.817	16,04	49,13

Gate	Number	%Total	%Gated
All	48.817	16,04	100,00
CD3+CD56-	47.689	15,67	97,69

Gate	Number	%Total	%Gated
All	47.689	15,67	100,00
NKG2D+ in CD3+CD56-	15.410	5,06	32,31

Supplementary file 1: autofluorescence control for NKG2A^{+}CD3^{+}CD56^{-} and NKG2D^{+}CD3^{+}CD56^{-} populations

Autofluorescence determination obtained with a blood sample unstained and processed as the stained blood sample

1. NKG2A^{+}CD3^{+}CD56^{-}

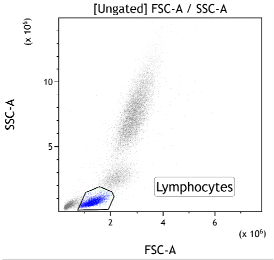

Gate	Number	%Total
All	38.458	100,00
Lymphocytes	8.828	22,95

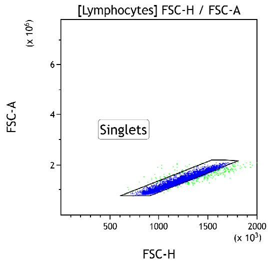

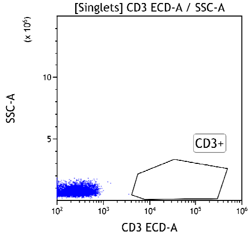

Gate	Number	%Total	%Gated
All	8.539	22,20	100,00
CD3+	0	0,00	0,00

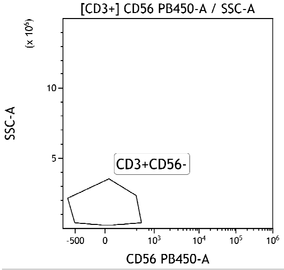

Gate	Number	%Gated
All	0	0,00
CD3+CD56-	0	N/A

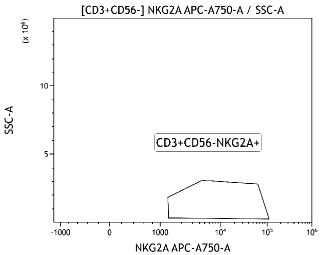

Gate	Number	%Total	%Gated
All	0	0,00	N/A
CD3+CD56-NKG2A+	0	0,00	N/A

2. NKG2D^{+}CD3^{+}CD56^{-}

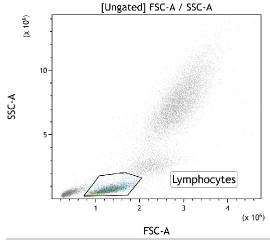

Gate	Number	%Total	%Gated
All	38.458	100,00	100,00
Lymphocytes	8.917	23,19	23,19

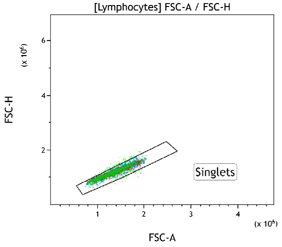

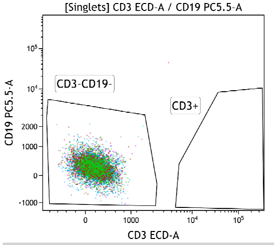

Gate	Number	%Total	%Gated
All	8.823	22,94	100,00
CD3-CD19-	8.822	22,94	99,99
CD3+	0	0,00	0,00

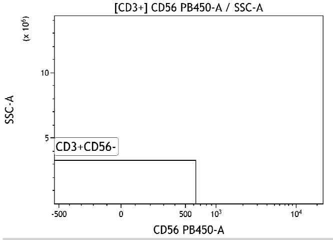

Gate	Number	%Total	%Gated
All	0	0,00	0,00
CD3+CD56-	0	0,00	N/A

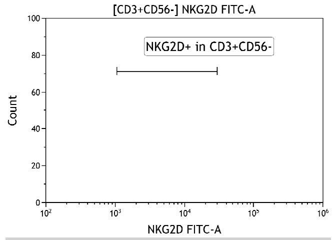

Gate	Number	%Total	%Gated
All	0	0,00	N/A
NKG2D+ in CD3+CD56-	0	0,00	N/A
