# Supplementary material for: Alterations in the immune system persist after one year of convalescence in severe COVID-19 patients
Source: Front Immunol. 2023 Feb 13;14:1127352. doi: 10.3389/fimmu.2023.1127352 (PMC9969554; doi:10.3389/fimmu.2023.1127352)
Supplement: Supplementary file 1 [file DataSheet_1.zip › Supplementary tables.docx]

Supplementary table 1.-Participants matched by sex, age and ethnic origin. CMC/CSC: convalescent mild/severe COVID-19 subjects. LA: Latin American; ES: European Spaniard.

| **CSC** | | | | | | **CMC** | | | **Controls** | | | |
| --- | --- | --- | --- | --- | --- | --- | --- | --- | --- | --- | --- | --- |
| **ID** | **Ethnic origin** | **Age** | **Sex** | **Months from ICU** | **ID** | **Ethnic origin** | **Age** | **Sex** | **ID** | **Ethnic origin** | **Age** | **Sex** |
| **S1** | LA | 31 | F | 12 | **M1** | LA | 30 | F |  |  |  |  |
| **S2** | ES | 67 | M | 12 | **M2** | ES | 67 | M | **C1** | ES | 65 | M |
| **S3** | ES | 60 | M | 11 | **M3** | ES | 61 | M |  |  |  |  |
| **S4** | LA | 40 | M | 11 | **M4** | LA | 39 | M | **C2** | ES | 40 | M |
| **S5** | ES | 52 | M | 11 | **M5** | ES | 51 | M | **C3** | ES | 51 | M |
| **S6** | ES | 63 | F | 10 | **M6** | ES | 64 | F | **C4** | ES | 60 | F |
| **S7** | ES | 66 | F | 11 | **M7** | LA (ES descent) | 71 | F | **C5** | ES | 64 | F |
| **S8** | ES | 55 | M | 14 | **M8** | ES | 52 | M |  |  |  |  |
| **S9** | ES | 47 | M | 13 | **M9** | ES | 50 | M | **C6** | ES | 51 | M |
| **S10** | ES | 60 | F | 15 | **M10** | ES | 59 | F | **C7** | ES | 55 | F |

Supplementary table 2.-Unmatched participants. Participants in the CSC group were unmatched by ethnic origin. Before inclusion in the general analysis, participants were stratified by ethnic origin and compared, and the global analysis was presented when the stratified comparison did not produce statistically significant results. Four CMC and two controls were redundant with matched participants or were matched by sex and age (and not ethnicity) with CSC. Information is included in the table. CMC/CSC: convalescent mild/severe COVID-19 subjects. LA: Latin American; ENS; European non Spaniard; M: Moroccan; ES: European Spaniard.

| **CSC** | | | | | **CMC** | | | | **Controls** | | | |
| --- | --- | --- | --- | --- | --- | --- | --- | --- | --- | --- | --- | --- |
| **ID** | **Ethnic**  **origin** | **Age** | **Sex** | **Months**  **from ICU** | **ID** | **Ethnic**  **origin** | **Age** | **Sex** | **ID** | **Ethnic origin** | **Age** | **Sex** |
| **S11** | LA | 53 | M | 12 |  |  |  |  |  |  |  |  |
| **S12** | LA | 56 | M | 5 |  |  |  |  |  |  |  |  |
| **S13** | LA | 47 | F | 12 |  |  |  |  | **C8** | ES | 41 | F |
| **S14** | ENS | 57 | M | 11 |  |  |  |  |  |  |  |  |
| **S15** | ES | 55 | M | 11 |  |  |  |  |  |  |  |  |
| **S16** | LA | 61 | F | 10 |  |  |  |  |  |  |  |  |
| **S17** | M | 57 | M | 11 |  |  |  |  |  |  |  |  |
| **S18** | LA | 50 | M | 4 |  |  |  |  |  |  |  |  |
|  |  |  |  |  | **M11** | ES | 47 | M |  |  |  |  |
|  |  |  |  |  | **M12** | ES | 64 | M |  |  |  |  |
|  |  |  |  |  | **M13** | ES | 53 | F | **C9** | ES | 56 | F |
|  |  |  |  |  | **M14** | ES | 54 | M |  |  |  |  |
